# Bergapten Improves Scopolamine-Induced Memory Impairment in Mice *via* Cholinergic and Antioxidative Mechanisms

**DOI:** 10.3389/fnins.2020.00730

**Published:** 2020-08-06

**Authors:** Joanna Kowalczyk, Łukasz Kurach, Anna Boguszewska-Czubara, Krystyna Skalicka-Woźniak, Marta Kruk-Słomka, Jacek Kurzepa, Małgorzata Wydrzynska-Kuźma, Grażyna Biała, Adrianna Skiba, Barbara Budzyńska

**Affiliations:** ^1^Chair and Department of Applied Pharmacy, Medical University of Lublin, Lublin, Poland; ^2^Independent Laboratory of Behavioral Studies, Medical University of Lublin, Lublin, Poland; ^3^Chair and Department of Medicinal Chemistry, Medical University of Lublin, Lublin, Poland; ^4^Chair and Department of Pharmacognosy with Medicinal Plant Unit, Medical University of Lublin, Lublin, Poland; ^5^Chair and Department of Pharmacology and Pharmacodynamics, Medical University of Lublin, Lublin, Poland

**Keywords:** acetylcholine, oxidative stress, coumarins, memory, passive avoidance

## Abstract

Bergapten is a furanocoumarin naturally occurring in the *Apiaceae* family *and* it is a well-known photosensitizing agent used in photochemotherapy. In this study, we investigated the influence of bergapten on cognitive function and mechanism underlying these effects in scopolamine-induced memory impairment in male Swiss mice. The passive avoidance test was used to evaluate the efficiency of memory acquisition and consolidation. The results demonstrated that both single and repeated administration of bergapten improved not only the acquisition but also consolidation of memory. The behavioral tests showed that bergapten prevented memory impairment induced by administration of scopolamine. Observed effects may result from the inhibition of acetylcholinesterase activity in the hippocampus and prefrontal cortex. Also, bergapten caused significant anti-oxidative effects. These new findings provide pharmacological and biochemical support for the development of the coumarin’s potential in cognitive deficits.

## Introduction

Dementia, a group of symptoms with memory deficits and losing social abilities, have become increasingly significant worldwide. They are especially problematic in developed countries. Memory loss in dementia has different causes, e.g., alteration of cholinergic transmission, oxidative stress, inflammation, or monoaminergic disturbances. These effects contribute to neuronal apoptosis and therefore to memory impairments (Deng et al., 2019). Alzheimer’s disease (AD) is considered as the most common cause of dementia in older people. Increased level of acetylcholinesterase (AChE) is one of the mechanisms underlying cognitive dysfunction in AD patients. This enzyme is responsible for acetylcholine (ACh) degradation. ACh is a neurotransmitter crucial for memory and learning processes. The increased amount of AChE is a consequence of AD rather than the main cause of the disease. AD patients experience neurodegeneration of cholinergic neurons especially in the basal nucleus of Meynert [responsible for about 90% ACh production in the central nervous system (CNS)], cerebral cortex, hippocampus, and amygdala (Wszelaki, 2009; Budzyñska et al., 2015). As a result, the level of ACh decreases, and neuronal transmission is ameliorated. Also, amyloid-beta (Aβ) aggregates cumulated in the brain can increase the production of AChE. Furthermore, the more advanced the disease is, the more butyrylcholinesterase (BChE) is produced. BChE is another form of cholinesterase and catalyzes the hydrolysis of ACh. When neuronal cells are destroyed, the level of BChE automatically becomes higher (Bukowska et al., 2017).

Acetylcholinesterase inhibitors (AChEIs) increase the level of ACh causing improvement of patient’s learning and memory functions. Procognitive medicines are commonly prescribed to people with AD. However, the use of AChEIs is limited by their hepatotoxicity and the side effects resulting from activation of the cholinergic system (Deng et al., 2019). The other limitation is the low bioavailability of the drug. However, cognitive functions are dependent not only on the level of ACh in the human brain but also on other neurotransmitters and factors (Parihar and Brewer, 2010).

It is well established that the CNS is especially susceptible to oxidants due to its specific construction and functions, such as high consumption of oxygen with a simultaneously high content of unsaturated lipids and weak antioxidant protection (Kruk-Slomka et al., 2012). Therefore, reactive oxygen species (ROS) overproduced in CNS cause dysfunction in membrane fluidity and decrease membrane potential, which in turn increases calcium permeability, especially in the regions of the brain such as the hippocampus, substantia nigra, and the striatum (Phaniendra et al., 2015). The oxidative mechanisms and affected brain regions are strictly connected with the pathology of several neurodegenerative diseases, including AD. Moreover, the mechanism underlying AD is also associated with increased ROS production, due to transition metal ions chelation [Cu, Zn, and Fe (III)] by neurofibrillary tangles. Aβ plaques, which are electron donors in Fenton or Haber–Weiss reactions can also be the source of ROS (Phaniendra et al., 2015). Therefore, antioxidant therapy is recommended as preventive care and effective support for the main therapy.

The current study is a continuation of our previous research regarding the activity of furanocoumarins in CNS (Budzyñska et al., 2015, 2016; Skalicka-Wozniak et al., 2018). Previously, we revealed the procognitive activity of xanthotoxin [8-methoxypsoralen; 9-methoxy-7H-furo(3,2-g)chromen-7-one], as well as its ability to prevent memory deficits induced by scopolamine (Skalicka-Wozniak et al., 2018). In the present study bergapten (5-methoxypsoralen), a structural analog of xanthotoxin was used. Bergapten was isolated from plants belonging to *the Apiaceae* family *and* is a well-known photosensitizing agent used in photochemotherapy (Tanew et al., 1999). It also shows anticancer (Panno et al., 2012), antioxidative (Liu et al., 2012), and anti-inflammatory properties (Bose et al., 2011). It was shown that bergapten was also the most potent inhibitor of CYP3A4 (Ho et al., 2001). Our studies revealed that bergapten prolongs antidepressant and procognitive effects of nicotine (Budzyñska et al., 2016; Skalicka-Wozniak et al., 2016). Also bergapten causes the increase of Ach in the brain by inhibiting the BChE and AChE activity (Senol et al., 2011, 2015; Wszelaki et al., 2011; Rohini and Srikumar, 2014). Bergapten was shown to be an effective inhibitor of monoamine oxidase (MAO), which resulted in the antidepressant effect (Huong et al., 1999).

Taking into account all the above, the current study aimed to evaluate the influence of bergapten on consolidation and acquisition of memory processes impaired by scopolamine administration in male Swiss mice. Subsequently, the level of AChE was measured in the prefrontal cortex and hippocampus, the structures important for cognitive functions, to check the mechanism underlying bergapten activity in memory impairments. Finally, to find out if the antioxidant mechanism is also engaged in neuroprotective action of bergapten, the oxidative stress markers in the aforementioned brain structures were determined.

## Materials and Methods

### Animals

Male Swiss mice weighing 20–25 g were maintained under the conditions of 12 h light/dark cycle, room temperature 21 ± 1°C, no limited tap water and laboratory chow (Agropol, Poland). Animals were becoming accustomed to the laboratory environment for one week. Experimental groups had 8–10 individuals. The National Institute of Health Guidelines for the Care and Use of Laboratory Animals and to the European Community Council Directive for the Care and Use of Laboratory Animals of September 22, 2010 (2010/63/EU) was kept during the whole experiment. The study was approved by the 1st Local Ethics Committee, Lublin, Poland. Different mice were used for each drug treatment.

### Drugs

Bergapten (5-methoxypsoralen) was extracted from fruits of *Heracleum leskovii* L using the method described previously by Budzyñska et al. (2016). The solution of scopolamine (Sigma-Aldrich, St. Louis, MO, United States) was prepared using 0.9% NaCl. The suspension of bergapten in a1% solution of Tween 80 (Sigma-Aldrich, St. Louis, MO, United States) and 0.9% NaCl solution was prepared as described previously by Budzyñska et al. (2016). Solutions were prepared daily and injected intraperitoneally (i.p., 10 ml/kg) Control groups were injected with an equal volume of the 0.9% NaCl solution.

### Experimental Procedure

The doses of bergapten and scopolamine were chosen based on literature data (Luszczki et al., 2010; Zhou et al., 2017), our recently published articles (Budzyñska et al., 2016), as well as on preliminary studies.

#### The Task for the Assessment of Memory-Related Responses

Memory-related responses were measured by the passive avoidance (PA) task (Venault et al., 1986). The apparatus used for the PA task comprized of an acrylic box divided into two compartments: a light compartment (10 × 13 × 15 cm) and a dark compartment (25 × 20 × 15 cm). The procedure was applied according to the description of Allami et al. (2011) and Javadi-Paydar et al. (2012) with minor modifications.

The experiment consisted of a pre-test and test. As was described previously by Budzyñska et al. (2016), in the pre-test after 30 s of habituation in the light compartment, the door was open and mice were allowed to enter into the dark space. Subsequently, the door was closed and the impulse of 0.15 mA (2 s) was generated. On the second day, the test was performed according to the same procedure but without electroshock. The time from opening the door to the entrance into the dark room was measured (pre-test – TL1, test – TL2). Depending on the used procedure, the time of drug administration, and the period between training and the test, PA allows examining different stages of memory. Drug administration before the first trial (pretest) should affect the acquisition of information, while drug administration immediately after the pretest should affect the consolidation of information.

#### Spontaneous Locomotor Activity

For spontaneous locomotor activity evaluation, an Opto-Varimex-4 Auto-Track (Columbus Instruments, United States) was chosen and used according to the procedure of Budzyñska et al. (2016). After subsequent injections of saline, bergapten (12.5, 25 mg/kg), scopolamine (1 mg/kg) or saline, or bergapten (12.5, 25 mg/kg) co-administered with scopolamine mice were placed separately in the apparatus for 15 min.

#### Rota-Rod Procedure

The ability of mice to maintain balance on a spinning rod was tracked by the Rota-rod procedure (Dunham and Miya, 1957). An animal was placed on a metal rod spinning at a constant velocity of 18 rpm. The parameter measured during the test was the period of time the mouse can stay on the spinning rod. However, a time limitation of 60 s per session applies.

Each experiment was preceded by a 3-min training session during which the mouse had an unlimited number of trails. Those rodents which were capable of maintaining balance the whole duration of the experiment passed the test.

#### Treatment

The first step of the experiment was designed to estimate the influence of an acute and sub-chronic administration of bergapten on the acquisition and consolidation of memory in mice, using the PA test.

During the acute treatment bergapten (12.5, 25, 50, and 100 mg/kg, i.p.) or saline (control group) were administered 30 min before the first trial (memory acquisition) or immediately after the first trial (memory consolidation) and re-tested after 24 h.

During the sub-chronic administration bergapten (12.5 and 25 mg/kg, i.p.) or saline (control group) were administered twice daily (8.00 a.m., 8.00 p.m.) for 6 days. On the seventh day bergapten (12.5 and 25 mg/kg, i.p.) or saline were administered only once (8.00 a.m), 30 min before the first trial (memory acquisition) or immediately after the first trial (memory consolidation) and re-tested after 24 h.

The next step of experiment was to evaluate the impact of an acute and sub-chronic administration of bergapten on the memory impairment induced by an acute administration of scopolamine in mice, using the PA test.

During the acute treatment bergapten (25 mg/kg, i.p.) or saline (control group) were administered 10 min before injection of scopolamine (1 mg/kg, i.p.) or saline. Pre-test was conducted 20 min after scopolamine administration (memory acquisition) or before appropriate injections (memory consolidation).

During the sub-chronic administration bergapten (12.5 mg/kg, i.p.) or saline (control group) were administered twice daily (8.00 a.m., 8.00 p.m.) for 6 days. On the seventh day bergapten (12.5 mg/kg, i.p.) or saline were administered only once (8.00 a.m) 10 min before saline or scopolamine (1 mg/kg) injection. Twenty minutes after scopolamine or saline administration (memory acquisition) or before appropriate injections (memory consolidation) the pre-test was conducted. Twenty-four hours later the mice were re-tested.

### Biochemical Procedures

#### Brain Tissue Dissection and Preparation

Immediately after the behavioral tests, mice were decapitated. As soon as the whole brains were taken out, they were immediately flushed with ice-cold saline to remove excessive blood. Then prefrontal cortex and hippocampus were resected. The isolated structures were homogenized in ice-cold saline for AChE determination or in 10% Tris buffer (pH 7.4) on ice for determination of oxidative stress markers. The homogenates were centrifuged at 10,000 *g* to separate nuclear debris.

#### Measurement of Acetylcholinesterase Activity

The activity of AChE was carried out using mouse AChE ELISA Kit from MyBio Source (MBS260553) according to the manufacturer’s instructions. The absorbance was read at 412 nm. The intensity of obtained color was proportional to the activity of AChE in the sample and it was expressed as enzymatic activity units (U).

#### Measurement of Total Antioxidant Capacity – TAC

Total antioxidant capacity (TAC) of tissue homogenates was determined spectrophotometrically by ferric-reducing ability of plasma (FRAP) method with modifications for tissue homogenates supernatants. A chromophore was produced by addition of 200 μl of working reagent [acetate buffer (pH 3.6), 2,4,6-tri-pyridyl-s-triazine (10 mmol/l) in 40 mmol/l HCl and aqueous solution of FeCl_3_ (20 mmol/l) in the ratio of 10:1:1] to 10 μl of samples diluted with 20 μl of deionized water at 96-well plate. The absorbance was measured at 593 nm after 30 min at 37°C and the results were evaluated from the standard curve prepared of FeSO_4_ at concentrations from 0 to 1000 μmol/l. The experiment was performed in triplicate and the final results are mean values of them.

### Statistical Analysis

The statistical analysis were performed using one- or two-way ANOVA for the factors of pretreatment, treatment and pretreatment/treatment interactions.

*Post hoc* comparison of means was carried out with the Tukey’s test (for one-way ANOVA) or with the Bonferroni’s test (for two-way ANOVA, scopolamine pretreatment, bergapten treatment and interaction between scopolamine and bergapten administration) for multiple comparisons, when appropriate. The data were considered statistically significant at confidence limit of *p* < 0.05. ANOVA analysis with Tukey’s or Bonferroni’s post tests were performed using GraphPad Prism version 5.00 for Windows, GraphPad Software, San Diego California USA^1^.

The results obtained in **the spontaneous locomotor activity** test were presented as an arithmetic average distance (cm) covered by a mouse ± SEM for each experimental group.

**For the evaluation of memory-related behavior**, a latency index (LI) was calculated as a difference between entrance latencies (TL2 and TL1) were calculated and depicted as a ratio:

LI=TL2-TL1/TL1

TL1 – the time taken to enter the dark compartment during the training; TL2 – the time taken to re-enter the dark compartment during the retention (Chimakurthy and Talasila, 2010).

## Results

### The Influence of an Acute Administration of Bergapten on the Memory Acquisition and Consolidation in the PA Test in Mice

#### Acquisition of Memory

**FIGURE 1:**
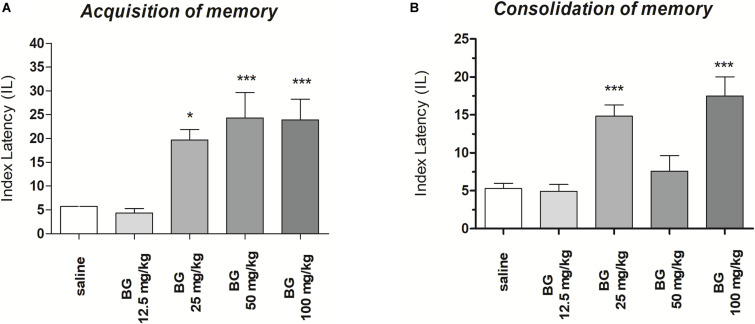
Effects of an acute bergapten or saline administration on the latency index (LI) during the acquisition trial **(A)** and consolidation trial **(B)**, using the PA test in mice. Bergapten (12.5, 25, 50, and 100 mg/kg, i.p.) or saline were injected 30 min before the first trial **(A)** or immediately after first trial **(B)** and mice were re-tested 24 h later; *n* = 8–10; the means ± SEM; *p < 0.05, ***p < 0.001 vs. saline-treated control group; Tukey’s test.

One-way ANOVA revealed that administration of acute i.p. doses of bergapten (12.5, 25, 50, and 100 mg/kg) had a statistically significant effect on LI values for memory acquisition [*F*(4,43) = 18.09; *p* < 0.0001). The *post hoc* Tukey’s test confirmed that the treatment with bergapten (25, 50, and 100 mg/kg) significantly increased LI values in mice compared to those in the saline-treated control group (*p* < 0.001) (Figure 1A), indicating that bergapten at these used doses improved acquisition of memory and learning.

#### Consolidation of Memory

One-way ANOVA revealed that administration of acute i.p. doses of bergapten (12.5, 25, 50, and 100 mg/kg) had a statistically significant effect on LI values for memory acquisition [*F*(4,48) = 14.45; *p* < 0.0001). The *post hoc* Tukey’s test confirmed that the treatment with bergapten significantly increased LI values in mice compared to those in the saline-treated control group [*p* < 0.001 – for the doses of bergapten: 25 and 100 mg/kg (Figure 1B)], indicating that bergapten, at these used doses, improved consolidation of memory and learning.

### The Influence of Sub-Chronic Administration of Bergapten on the Memory Acquisition and Consolidation in the PA Test in Mice

#### Acquisition of Memory

**FIGURE 2:**
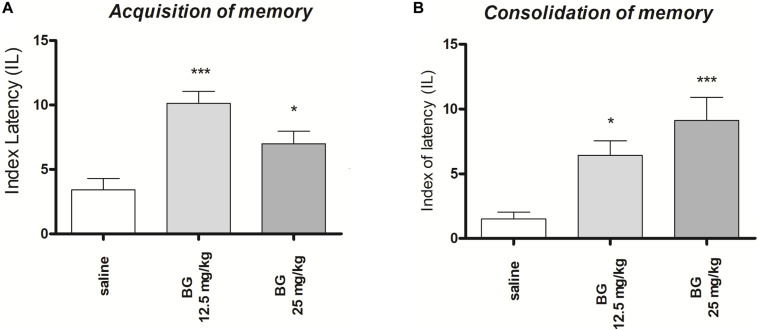
Effects of sub-chronic bergapten or saline administration on the latency index (LI) during the acquisition trial **(A)** and consolidation trial **(B)**, using the PA test in mice. Bergapten (12.5 and 25 mg/kg, i.p.) or saline were administered for the 6 days. On the seventh day bergapten was administered 30 min before the first trial **(A)** or immediately after first trial **(B)** and mice were re-tested 24 h later; *n* = 8–10; the means ± SEM; *p < 0.05; ***p < 0.001 vs. saline-treated control group; Tukey’s test.

One-way ANOVA revealed that administration of both sub-chronic i.p. doses of bergapten (12.5 and 25 mg/kg) had a statistically significant effect on LI values for memory acquisition [*F*(2,19) = 11.86; *p* = 0.0005]. The *post hoc* Tukey’s test confirmed that the treatment with bergapten significantly increased LI values in mice compared to those in the saline-treated control group (*p* < 0.001 – for the dose of 12.5 mg/kg and *p* < 0.05 for the dose of 25 mg/kg (Figure 2A), indicating that bergapten, at the used doses, improved acquisition of memory and learning.

#### Consolidation of Memory

One-way ANOVA revealed that administration of both sub-chronic i.p. doses of bergapten (12.5 and 25 mg/kg) had a statistically significant effect on LI values for memory acquisition [*F*(2,24) = 9.986; *p* = 0.0007]. The *post hoc* Tukey’s test confirmed that the treatment with bergapten significantly increased LI values in mice compared to those in the saline-treated control group (*p* < 0.05 – for the dose of 12.5 mg/kg and *p* < 0.001 for the dose of 25 mg/kg (Figure 2B), indicating that bergapten, at these used doses, improved acquisition of memory and learning.

### The Influence of an Acute Administration of Bergapten on the Memory Impairment Induced by an Acute Injection of Scopolamine

#### Acquisition of Memory

**FIGURE 3:**
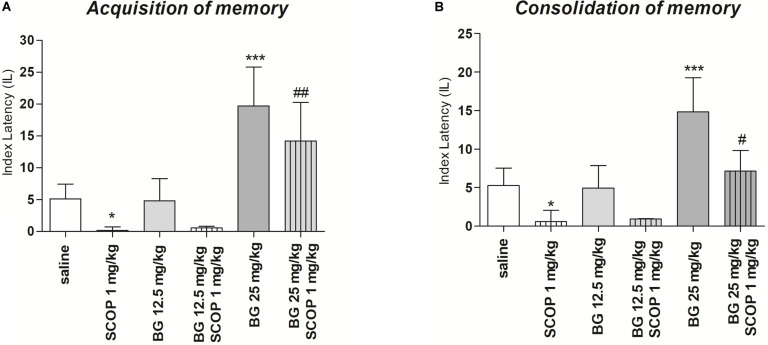
Effects of acute administration of bergapten (BG) on scopolamine-induced impairment of memory acquisition trial **(A)** and consolidation trial **(B)** using the PA test in mice. Bergapten (12.5 and 25 mg/kg, i.p.) was administered 30 min and scopolamine (SCOP, 1 mg/kg, i.p.) 20 min before the first trial **(A)** or bergapten was administered immediately after first trial and scopolamine 10 min after bergapten injection **(B)** and animals were re-tested 24 h after the last injection; *n* = 8–12; the means ± SEM; *p < 0.05; ***p < 0.001 vs. saline-treated control group, ^#^*p* < 0.05, ^###^*p* < 0.01 vs. scopolamine-treated group; Bonferroni’s test.

For memory acquisition, two-way ANOVA analyses revealed that there was statistically significant effect caused by bergapten (25 mg/kg) pretreatment [*F*(1,51) = 1.90; *p* = 0.1745] as well as by scopolamine (1 mg/kg) treatment [*F*(2,51) = 7,08; *p* = 0.0019]) but there is no statistically significant effect caused by interactions between bergapten pretreatment and scopolamine treatment [*F*(2,51) = 3.81; *p* = 0.0287]. The *post hoc* Bonferroni’s test confirmed that scopolamine at the dose of 1 mg/kg significantly decreased LI values in mice in the PA test in comparison to the saline/saline-treated mice, suggesting the amnesic effect of this drug (*p* < 0.05). The *post hoc* Bonferroni’s test also confirmed that bergapten, at these doses of 25 mg/kg, improved acquisition of memory and learning (*p* < 0.001). Additionally, bergapten (25 mg/kg) attenuated this amnesic effect of scopolamine (1 mg/kg) (*p* < 0.05) as compared with scopolamine-treated (1 mg/kg) mice (Figure 3A).

### Consolidation of Memory

For memory consolidation, two-way ANOVA analyses revealed that there was statistically significant effect caused by bergapten pretreatment [*F*(1,32) = 7.02; *p* = 0.012, *F*(2,51) = 7.08; *p* = 0.00194] as well as by interactions between bergapten pretreatment and scopolamine treatment [*F*(2,51) = 3.81; *p* = 0.0287] but there is no statistically significant effect caused by scopolamine (1 mg/kg) treatment [*F*(1,51) = 1.90; *p* = 0.1745]. The *post hoc* Bonferroni’s test showed that scopolamine at the dose of 1 mg/kg significantly decreased LI values in mice in the PA test in comparison to the saline-treated mice, suggesting the amnesic effect of this drug (*p* < 0.05). The *post hoc* Bonferroni’s test also confirmed that bergapten, at these doses of 25 mg/kg, improved consolidation of memory and learning (*p* < 0.001). Additionally, bergapten (25 mg/kg) attenuated this amnesic effect of scopolamine (1 mg/kg) (*p* < 0.05) as compared with scopolamine-treated (1 mg/kg) mice (Figure 3B).

### Repeated Bergapten Injection Effects on Memory-Related Processes Induced by Scopolamine in the PA Test in Mice

**FIGURE 4:**
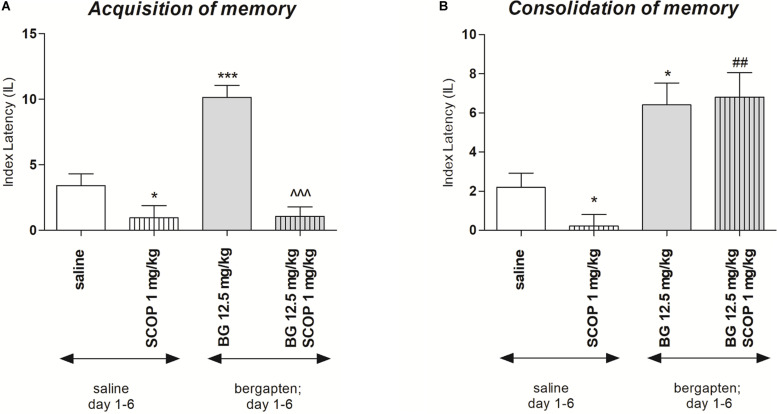
Effects of subchronic administration of bergapten (BG) on scopolamine-induced impairment of memory acquisition trial **(A)** and consolidation trial **(B)** using the PA test in mice. Bergapten (12.5 mg/kg, i.p.) was administered 6 days twice daily. On the seventh day bergapten was administered 30 min and scopolamine (SCOP, 1 mg/kg, i.p.) 20 min before the first trial **(A)** or bergapten was administered immediately after first trial and scopolamine 10 min after bergapten injection **(B)** and animals were re-tested 24 h after the last injection; *n* = 8–12; the means ± SEM; *p < 0.05; ***p < 0.01 vs. saline-treated control group, ^##^*p* < 0.01 vs. scopolamine-treated control group; ^^^*p* < 0.001 vs. bergapten-treated (12.5 mg/kg) group; Bonferroni’s test.

In order to check the influence of subchronic bergapten administration on acquisition and consolidation of the memory processes the doses of 12.5 mg/kg as the inactive in PA test was selected. Figure 4A indicates the effects of repeated injections of bergapten on memory acquisition impaired by scopolamine during the retention trial in the PA task [two-way ANOVA: pre-treatment (*F*(1,32) = 14.70; *p* = 0.0006), treatment (*F*(1, 32) = 41.77; *p* < 0.0001) and interactions effect (*F*(1, 32) = 13.74; *p* = 0.0008)]. The *post hoc* Bonferroni’s test revealed that bergapten given repeatedly, at the doses of 12.5 mg/kg significantly increased IL value, as compared with the saline-treated mice, thus indicating that subchronic administration of bergapten improved acquisition of the memory and learning processes during the retention trial (*p* < 0.001). In contrast, when mice were treated subchronically with saline and with scopolamine (1 mg/kg) on the seventh day, the impairment of memory acquisition was observed (*p* < 0.05) as compared with saline-treated group. Furthermore, we did not observe improvement in memory and learning processes in the animals injected repeatedly with bergapten (12.5 mg/kg). Also on the seventh day of injection, we did not observe improvement in memory and learning processes in animals injected with bergapten in combination with scopolamine when compared to the mice treated exclusively with scopolamine.

The changes of IL values, indicated by two-way ANOVA, were noticed at the consolidation trial [treatment (*F*(1,30) = 1.64; *p* = 0.2107), and without pre-treatment effect (*F*(1,30) = 33.91; *p* < 0.0001) and interactions effect (*F*(1,30) = 1.64; *p* = 0.2107)] (Figure 4B). The *post hoc* Bonferroni’s test showed that repeated injection of bergapten (12.5 mg/kg) improved the cognitive processes (*p* < 0.001), compared to the saline-treated mice. Additionally, mice subchronically treated with saline and on the seventh day with scopolamine, exhibited the impairment of memory consolidation (*p* < 0.05). It was also revealed that repeated administration of bergapten at the doses of 12.5 mg/kg significantly decreased impairment of memory and learning processes induced by an acute injection of scopolamine (*p* < 0.01), as compared with subchronically saline-treated group and on the seventh day scopolamine-treated mice (Figure 4b).

### Effect of Acute Administration of Bergapten on Motor Coordination in Rota-Rod in Mice

In the rota-rod test no changes in motor coordination were observed [one-way ANOVA (*F*(3,26) = 3.067; *p* = 0.0536)]. Value on time on rotating rod (s) ± SEM: vehicle 60.00 ± 0.00; bergapten 12.5 mg/kg 58.25 ± 1.750; bergapten 25 mg/kg 60.00 ± 0.00; bergapten 50 mg/kg 50.71 ± 4.91

### Single Injection of Bergapten, Scopolamine and Co-administration of Both Drugs on Locomotor Activity in Mice

Table 1 shows the influence of an acute administration of bergapten and scopolamine on locomotor activity. Two-way ANOVA analyses revealed that there was statistically significant effect caused by as well as by scopolamine (1 mg/kg) treatment [*F*(2,42) = 14.45; *p* = 0.0005] but there is no statistically significant effect caused by bergapten pretreatment [*F*(1,42) = 2.20; *p* = 0.1236] as well as interactions between bergapten pretreatment and scopolamine treatment [*F*(2,51) = 3.81; *p* = 0.0287]. The *post hoc* Bonferroni’s test confirmed that scopolamine at the dose of 1 mg/kg significantly increased locomotor activity in mice in comparison with the saline-treated mice (*p* < 0.01). The *post hoc* Bonferroni’s test also confirmed that co-administration of scopolamine with bergapten, at these doses of 12.5 mg/kg (*p* < 0.05) and 25 mg/kg (*p* < 0.01) increased locomotor activity as compared with bergapten-treated mice (Table 1). Bergapten at the doses of 50 mg/kg (458.10 ± 41.41) and 100 mg/kg (385.90 ± 42.15) mg/kg did not influenced on observed parameter when compared with saline-treated mice.

**TABLE 1:** Effect of bergapten (BG, 12.5, 25 mg/kg, i.p.) and scopolamine (SCOP, 1 mg/kg, i.p.) on spontaneous locomotor activity in mice.

	**Saline**	**SCOP**	**BG 12.5 mg/kg**	**BG 25 mg/kg**	**BG 12.5 mg/kg + SCOP**	**BG 25 mg/kg + SCOP**
Photocell beam breaks ± SEM (30 min)	402.30 ± 31.01	863.30 ± 188.70***	439.50 ± 30.31	398.10 ± 24.54	534.25 ± 68.45^###^	657.12 ± 56.19^$$$, ###^

*Drugs were administered separately or in combination. Animals were injected with bergapten 15 min before scopolamine administration and then immediately placed in actimeters for the 30 min. Data are presented as the means ± SEM. *n* = 8–12; ^∗∗∗^*p* < 0.001, vs. saline-treated group; ^###^*p* < 0.001 vs. scopolamine-treated group; ^$$$^*p* < 0.001, vs. bergapten (25 mg/kg)-treated group, Bonferroni’s test.*

### Biochemical Studies

**FIGURE 5:**
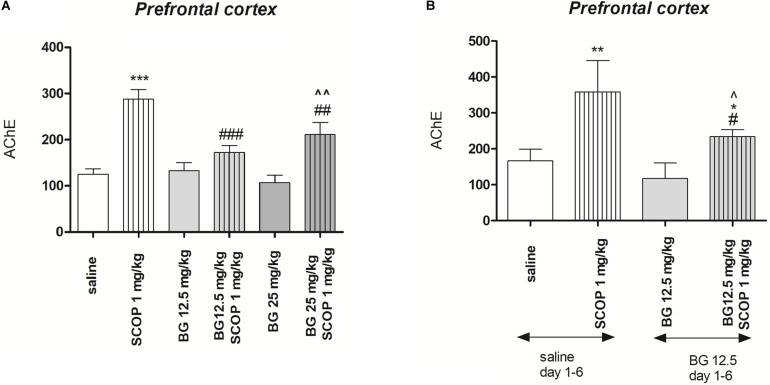
Effects of acute **(A)** or subchronic **(B)** administration of bergapten (BG) on AChE level increased by scopolamine administration in the prefrontal cortex in mice. BG was administered 30 min and scopolamine (SCOP, 1 mg/kg, i.p.) *n* = 8–12; the means ± SEM; *p < 0.05, **p < 0.01, ***p < 0.001 *vs.* saline-treated control group, ^#^*p* < 0.05, ^##^*p* < 0.01, ^###^*p* < 0.001 vs. scopolamine-treated control group, ^*p* < 0.05, ^^*p* < 0.01 vs. bergapten-treated group; Bonferroni’s test.

Figure 5A indicates the effects of bergapten (12.5 and 25 mg/kg) administered alone or in combination with scopolamine on AChE level measured in the prefrontal cortex [pretreatment (*F*(1,50) = 33.58, *p* = 0.0352), interactions effect (*F*(1,50) = 4.08, *p* = 0.0229), treatment (*F*(1,50) = 37.21, *p* < 0.0001); two-way ANOVA]. *Post hoc* Bonferroni’s test confirmed the significant increasing of the AChE concentration in the prefrontal cortex after single injection of scopolamine (*p* < 0.001). No changes were noticed after single acute injection of bergapten (12.5 and 25 mg/kg), however, combination of bergapten at both doses and scopolamine decreased the level of AChE in the prefrontal cortex (12.5 mg/kg – *p* < 0.001; 25 mg/kg – *p* < 0.01) in comparison with scopolamine-treated group (Figure 5A).

Figure 5B indicates the effects of subchronic bergapten (12.5 and 25 mg/kg) administered alone or in combination with acute injection of scopolamine on AChE level measured in the prefrontal cortex [pretreatment (*F*(2,35) = 24.28, *p* < 0.0001), interactions effect (*F*(1,35) = 4.51, *p* = 0.0409), treatment (*F*(1,35) = 76.40, *p* < 0.0001); two-way ANOVA]. *Post hoc* Bonferroni’s test confirmed the significant increase of the AChE concentration in the prefrontal cortex after single injection of scopolamine (*p* < 0.01). No changes were noticed after single subchronic injection of bergapten (12.5 mg/kg), however, we observed attenuation of enzyme level in the prefrontal cortex (*p <* 0.05) in comparison with scopolamine- and saline treated group (Figure 5B).

**FIGURE 6:**
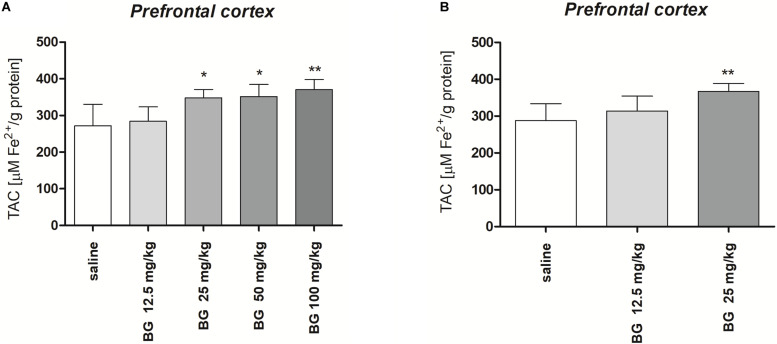
Total antioxidant capacity (TAC) of the prefrontal cortex of mice brain after single **(A)** or subchronic **(B)** administration of bergapten (BG). The results are expressed as micromoles per gram protein and presented as the means ± SD; *n* = 10; *p < 0.05, **p < 0.01 vs. saline-treated control group; Tukey’s test.

Figure 6A presents the effect of single dose of bergapten (12.5, 25, 50, 100 mg/kg) on TAC measured in the prefrontal cortex [*F*(4,25) = 7.791, *p* = 0.0003; one-way ANOVA]. *Post hoc* Tukey test confirmed significant increase in TAC level after single injection of bergapten in doses 25 and 50 mg/kg (*p <* 0.05) and 100 mg/kg (*p <* 0.01).

Figure 6B presents the effect of subchronic bergapten (12.5, 25 mg/kg) on TAC measured in the prefrontal cortex [*F*(2,15) = 6.794, *p* = 0.0079; one-way ANOVA]. *Post hoc* Tukey test confirmed significant increase in TAC level after single injection of bergapten in dose 25 mg/kg (*p <* 0.01) in comparison with saline-treated group.

**FIGURE 7:**
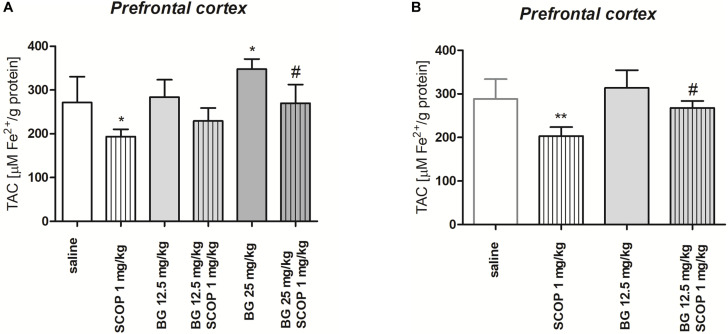
Total antioxidant capacity (TAC) of the prefrontal cortex of mice brain after single **(A)** or subchronic **(B)** administration of bergapten (BG) or saline with or without scopolamine (1 mg/kg) administered 1 h before the decapitation. The results are expressed as micromoles per gram protein and presented as the means ± SD; *n* = 10; *p < 0.05, **p < 0.01 vs. saline-treated control group; ^#^*p* < 0.05 vs. scopolamine-treated group; Bonferroni’s test.

Figure 7A indicates the effect of single dose of bergapten (12.5, 25 mg/kg) administered alone or in combination with scopolamine (1 mg/kg) on TAC determined in the prefrontal cortex [pretreatment (*F*(2,54) = 21.35, *p <* 0.0001), treatment (*F*(1,54) = 51.61, *p <* 0.0001) without interactions (*F*(2, 54) = 0.6146, *p* = 0.5446); two-way ANOVA]. *Post hoc* Tukey’s test confirmed statistically significant decrease in TAC level in the prefrontal cortex after single injection of scopolamine (*p <* 0.05). Administration of bergapten caused dose-dependent increase in TAC level, being statistically significant for the highest dose of bergapten (25 mg/kg, *p <* 0.05) in comparison to scopolamine-treated group (Figure 7A).

Figure 7B indicates the effect of subchronic bergapten (12.5 mg/kg) administered alone or in combination with scopolamine (1 mg/kg) on TAC determined in the prefrontal cortex [pretreatment (*F*(1,36) = 18.44, *p* = 0.0001), treatment (*F*(1,36) = 38.61, *p <* 0.0001) without interactions (*F*(1,36) = 3.433, *p* = 0.0721); two-way ANOVA]. *Post hoc* Tukey’s test confirmed statistically significant decrease in TAC level after single injection of scopolamine (*p* < 0.01). Administration of bergapten caused increase in TAC level, being statistically significant in the prefrontal cortex (*p* < 0.05) in comparison to scopolamine-treated group (Figure 7B).

## Discussion

In the present study, we revealed for the first time the procognitive effects of bergapten on memory processes, consolidation, and acquisition, in the scopolamine model of memory impairment in male Swiss mice. We also evaluated the presumable mechanisms underlying these effects. Our previous findings showed that xanthotoxin improved scopolamine-impaired consolidation and acquisition of memory. This effect resulted from decreased AChE activity and attenuation of oxidative stress processes (Skalicka-Wozniak et al., 2018). Consequently, in the present study, scopolamine was used as a tool substance for the induction of memory deficits in rodents. It was revealed that this compound induces cognitive disorders in various paradigms: radial maze (Buresová and Bures, 1982; Rahimzadegan and Soodi, 2018), object recognition and spatial tasks (Sambeth et al., 2007), Morris water maze, and PA test (Cozzolino et al., 1994). It is suggested that muscarinic receptors’ blockade is crucial for memory impairment induced by scopolamine. Also, (Jang et al., 2013) apoptosis (Jahanshahi et al., 2013) and inflammatory responses (Ahmad et al., 2014) in brain tissue may play a role in these effects. One of the mechanisms responsible for scopolamine-induced amnesia is oxidative stress. Scopolamine was reported to exert pro-oxidative effects as it decreased activities of antioxidant enzymes such as superoxide dismutase, catalase, and glutathione peroxidase (Uma and Maheswari, 2014), and increased the concentration of malondialdehyde (MDA), which is the main marker of lipids peroxidation (Abd-El-Fattah et al., 2014). Moreover, several studies proved procognitive effects of antioxidant compounds in scopolamine-induced memory impairment, probably through attenuating the markers of oxidative stress (Harrison et al., 2009; Hritcu et al., 2015). Therefore, oxidative stress seems to play a crucial role in memory deficits caused by scopolamine.

The PA test was used as a validated paradigm for evaluation of the influence of different compounds on memory processes. PA is considered to be an aversive conditioning paradigm in which rodents learn to associate a specific compartment with the appearance of an unpleasant/aversive stimulus (e.g., electric shock). It is well established that administration of scopolamine as a nonselective muscarinic receptor antagonist before PA training induces impairment of acquisition of memory processes, whereas administration of scopolamine immediately after PA training disrupts consolidation of memory processes (Skalicka-Wozniak et al., 2018). Subsequent studies showed that the administration of cholinomimetic drugs such as rivastigmine antagonized memory deficits induced by scopolamine (Bejar et al., 1999).

In our studies, we examined the acute and subchronic effects of bergapten. First, we evaluated if one dose of compound affects memory and learning processes. Subsequently, we assessed if any changes in behavioral processes and biochemical parameters occurred during subchronic administration. Since all of the current anti-dementia therapies are long-lasting, it was recommended to test the procognitive activity of bergapten also in the subchronic model of administration. The changes after acute and subchronic injections were compared with each other. Our behavioral study revealed that bergapten administered acutely at the doses of 25 mg/kg and 100 mg/kg improved acquisition as well as the consolidation of memory. Administered acutely at the dose of 50 mg/kg bergapten improved only the acquisition of memory. Furthermore, the coumarin at the lowest dose (12.5 mg/kg) did not show the influence on cognitive function. Subchronic administration improved both acquisition and consolidation (12.5 and 25 mg/kg) of memory processes, however, the effect is not dose-dependent. Additionally, acute administration of bergapten (25 mg/kg) decreased scopolamine-induced impairment of memory, whereas the dose of 12.5 mg/kg did not prevent amnesic effects of scopolamine. Furthermore, the subchronic administration of bergapten (12.5 mg/kg) improved only the consolidation of memory processes impaired by scopolamine. Interestingly, both acute and chronic injections of tested coumarin deceased the level of AChE elevated by scopolamine administration but TAC level was increased after the subchronic, not acute, model of drugs administration. Thus, we may speculate that cholinergic mechanisms (AChE level) together with antioxidative processes enhance procognitive effects induced by bergapten. However, other molecular and adaptive receptor changes may underlay these effects.

Importantly, bergapten at the used doses did not influence the motor coordination and locomotor activity. However, we cannot rule out adverse effects at other levels, such as anxiety, hepatotoxicity, or other characteristics for cholinomimetic drugs. Although we observed an increase of locomotor activity in the groups treated with scopolamine and bergapten (at both doses) when compared to scopolamine-injected mice, these results did not affect the acquisition and consolidation of memory as these processes were evaluated 24 h after the last injection. The next step in our study was to evaluate possible mechanisms underlying the procognitive effects of bergapten as well as the reason for the amelioration of cognitive deficits induced by scopolamine administration. Thus, the level of AChE and oxidative stress markers were examined. Biochemical analyses were performed on selected brain structures: the prefrontal cortex and hippocampus. The hippocampus is a part of the limbic system responsible for long-term and spatial memory. It is considered to be a structure that transfers information from short-term memory into long-term memory. It leads to the occurrence of various memories and learning processes (Abraham et al., 2019). Working (operational) memory is located in the prefrontal cortex (Anand and Dhikav, 2012). The cholinergic system is primarily involved in memory and attention-related functions. The age-related memory impairment, among others, results from a dysfunction of cholinergic transmission. ACh activates the neurons of the neocortex and hippocampus thereby facilitating the stimulation related to reward and associative learning (Rezayof et al., 2008; Yousefi et al., 2012; Zarrindast et al., 2012; Khakpai et al., 2013; Piri et al., 2013). ACh exerts its effects by activation of the muscarinic or nicotinic cholinergic receptors. Afterward, AChE and BuChE, enzymes synthesized in the postsynaptic membrane, break down ACh into choline and acetic acid. Physiologically, this mechanism prevents excessive activation of the neuronal system. However, the excessive degradation of ACh by AChE and BuChE leads to deterioration of cognitive functions. Thus, it is reasonable to introduce cholinesterase inhibitors as anti-dementia drugs. Many natural products or whole extracts from natural products inhibit AChE activity (Vallejo et al., 2007, 2009, 2017). Also, inhibitory activities of bergapten toward cholinesterase were investigated *in vitro*. It was revealed that bergapten, even at the lowest tested doses (12.5 μg/mL^–1^), inhibited BChE more than AChE (37.77 and 84.82%, respectively), which is even more potent than galantamine (98.97 and 80.31% at 100 μg/mL^–1^) (Senol et al., 2011).

Thus, our study was undertaken to evaluate bergapten activity as an AChE inhibitor in *ex vivo* study. We showed for the first time, that administration of bergapten either subchronically or acutely, did not change the level of AChE in the hippocampus and prefrontal cortex. In light of the in vitro experiments, further research concerning inhibition of BuChE should be undertaken *ex vivo*. Interestingly, bergapten diminished the level of the enzyme increased previously by scopolamine administration and these results comply with our previous study. Our previous experiments showed that acute and subchronic administration of xanthotoxin (1 mg/kg) did not influence the level of AChE in both tested brain structures, although it prevented the increase of the AChE activity caused by a single dose of scopolamine. Also, no improvement of memory and learning functions in the PA test after co-administration xanthotoxin and scopolamine was observed.

Memory improvement noticed after acute and subchronic administration of bergapten seems to be independent of AChE activity. Thus, other mechanisms may underlie these effects. On the other hand, bergapten decreased AChE activity when the level of the enzyme was altered by scopolamine application.

Additionally, as it was mentioned previously (Skalicka-Wozniak et al., 2018), processes connected with oxidative stress may underlie the improvement of cognitive function induced by coumarins. Our study proved that oxidative stress is involved in scopolamine-induced dementia as we observed a decrease in TAC as well as an increase in MDA (Supplementary Material) concentration. It can be the reason for long- and short-term memory’s interferences too. Furthermore, our study revealed antioxidant properties of bergapten as well as its antioxidant activity against scopolamine-induced oxidative stress in a single dose and subchronic models of administration. Interestingly, the prefrontal cortex was more affected by bergapten than the hippocampus. This effect probably comes from the specific functions of these regions in memory and cognitive functions. The prefrontal cortex is responsible for filtration, interpretation, and moderation of emerging stimuli (through sense organs) and thoughts that come into the brain. In other words, the prefrontal part is involved in slowing down aggression and fear, and blocking curiosity, which allows remembering emotions accompanying the events. The hippocampus is mainly responsible for declarative memory (regarding various events and situations), as well as for spatial memory and long-term memory. This means that it is in the “second line” of cognitive processes and probably it is less susceptible to temporary changes because it maintains its internal homeostasis (Tyng et al., 2017). The prefrontal cortex is also phylogenetically the youngest brain structure, therefore it is also the most susceptible to damage (Passingham and Wise, 2012). The coumarins can act as antioxidants to protect cells against free radicals.

Similarly to our findings, it was also revealed that Ishige foliacea extract (Kim et al., 2020) and *Elaeagnus umbellata* fruit extract improved memory processes in the scopolamine model of dementia in mice. Both of them showed antioxidant activity in the brain as well as AChE inhibition. Nazir et al. (2020) found out that *E. umbellata* fruit extract administered orally decreased the level of the highly active free radical, DPPH, in the hippocampus and frontal cortex. This antioxidant effect was associated with AChE inhibition and the procognitive effect in the novel object recognition test and Y-Maze test (Nazir et al., 2020). The study of Roshanbakhsh et al. (2020) revealed that another natural compound, piperine, improved memory impairments in rats by diminishing the iNOS level and increasing TAC in the hippocampus. This effect was associated with spatial memory improvement in the Morris water maze test (Roshanbakhsh et al., 2020). The scopolamine model of dementia was used by Ngoupaye et al. (2017) to prove the antioxidative effect of Gladiolus dalenii lyophilisate in the process of working and spatial memory improvement. In this study, the levels of malondialdehyde and AChE were decreased (Ngoupaye et al., 2017). We reported a similar result in our project. Due to the above, we suggest that antioxidant activity together with AChE inhibition in the hippocampus and prefrontal cortex might be significant in the improvement of memory processes.

Moreover, it was revealed that bergapten can inhibit MAO activity involved in regulation, e.g., depressive behaviors (Huong et al., 1999). This compound also shows anti-inflammatory properties associated with inhibition of interleukin 8, interleukin 6, and tumor necrosis factor (TNF). Oxidative stress and the neurotransmitter abnormalities underlie neurodegenerative diseases associated with memory impairments. Consequently, coumarins such as bergapten may become the basis for prophylaxis as well as therapies for these diseases (Budzyñska et al., 2015; Skalicka-Wozniak et al., 2018).

## Conclusion

In summary, bergapten might be able to improve the acquisition and consolidation of memory. At the same time, it can effectively alleviate the cognitive symptoms of scopolamine-induced amnesia in the mouse. The mechanism of these effects seems unrevealed as bergapten did not change the level of AChE by itself but decreased the level of enzyme changed as a consequence of scopolamine administration. These new findings provide pharmacological and biochemical support for the development of the potential of coumarin in cognitive deficits. This hypothesis will form a new direction for our future research. Also, further studies concerning the administration of bergapten after scopolamine injection, to observe whether this compound can reverse established cognitive deficits, and not only propose them as prophylactic agents should be considered.

## Data Availability Statement

The raw data supporting the conclusions of this article will be made available by the authors, without undue reservation.

## Ethics Statement

The animal study was reviewed and approved by Local Ethics Committee in Lublin, Poland.

## Author Contributions

JK, MW-K, and BB performed behavioral experiments. KS-W and AS performed phytochemical isolation. AB-C and JK performed biochemical study. JK, BB, MK-S, and AB-C analyzed data. KS-W, BB, AB-C, and JK wrote the manuscript. GB checked the manuscript. KS-W provided research materials. All authors contributed to the article and approved the submitted version.

## Conflict of Interest

The authors declare that the research was conducted in the absence of any commercial or financial relationships that could be construed as a potential conflict of interest.

## Footnotes

1 www.graphpad.com
