# Supplementary figures and images for: Bergapten Improves Scopolamine-Induced Memory Impairment in Mice via Cholinergic and Antioxidative Mechanisms
Source: Front Neurosci. 2020 Aug 6;14:730. doi: 10.3389/fnins.2020.00730 (PMC7438900; doi:10.3389/fnins.2020.00730)

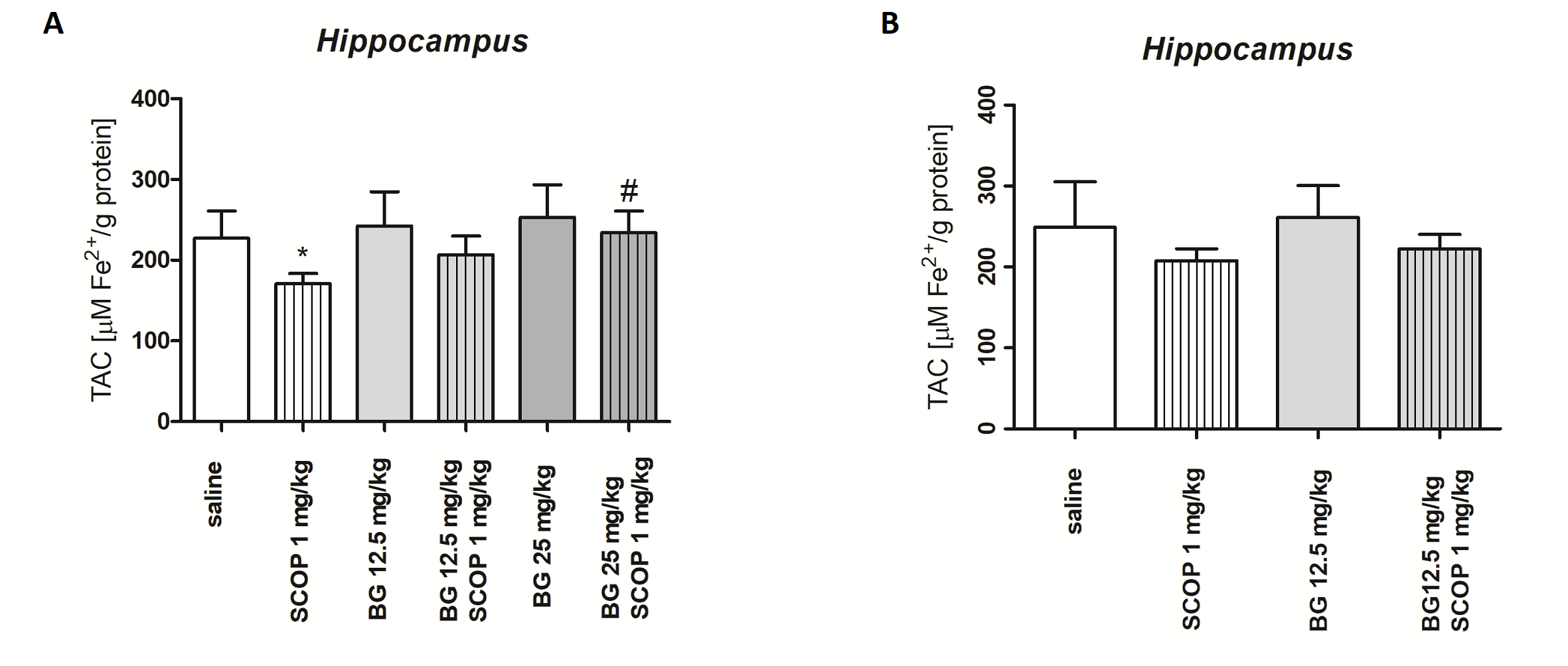

Supplement: Supplementary file 1 [file Image_1.TIF]

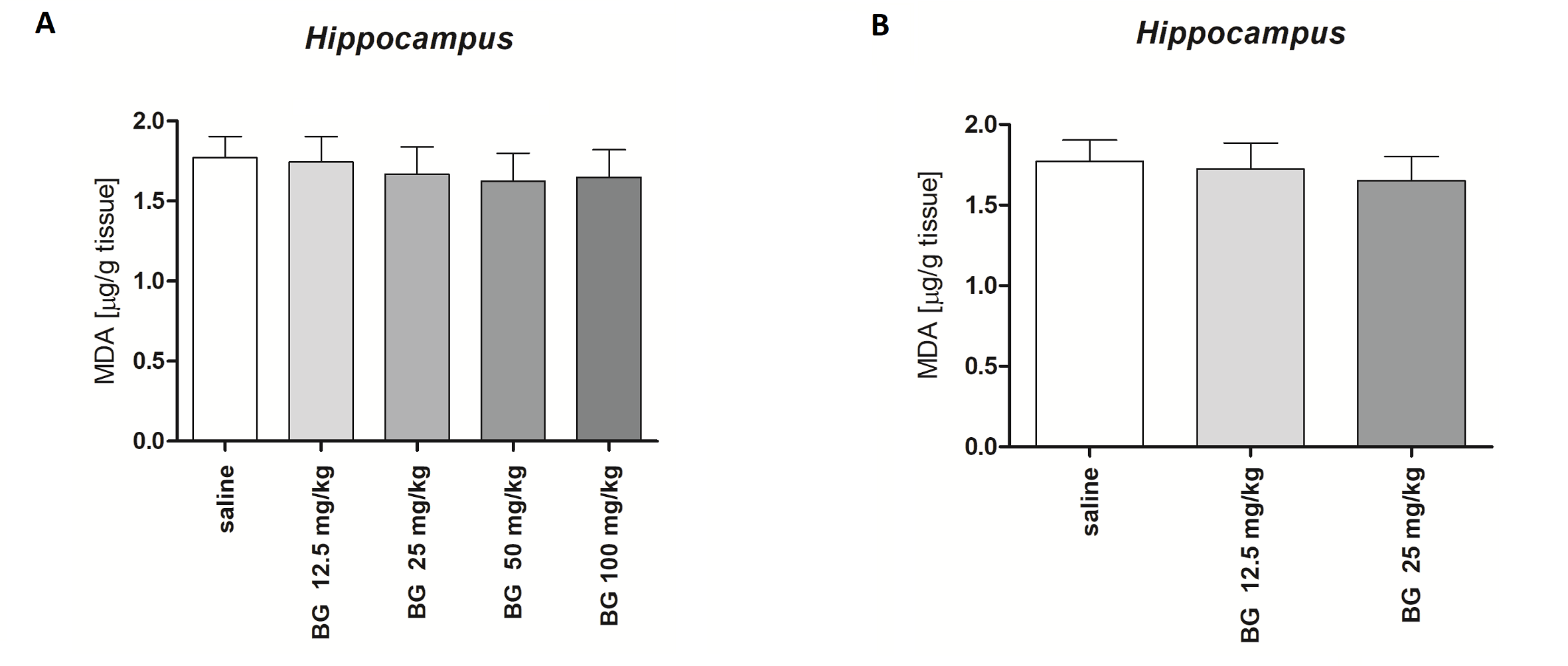

Supplement: Supplementary file 2 [file Image_2.TIF]

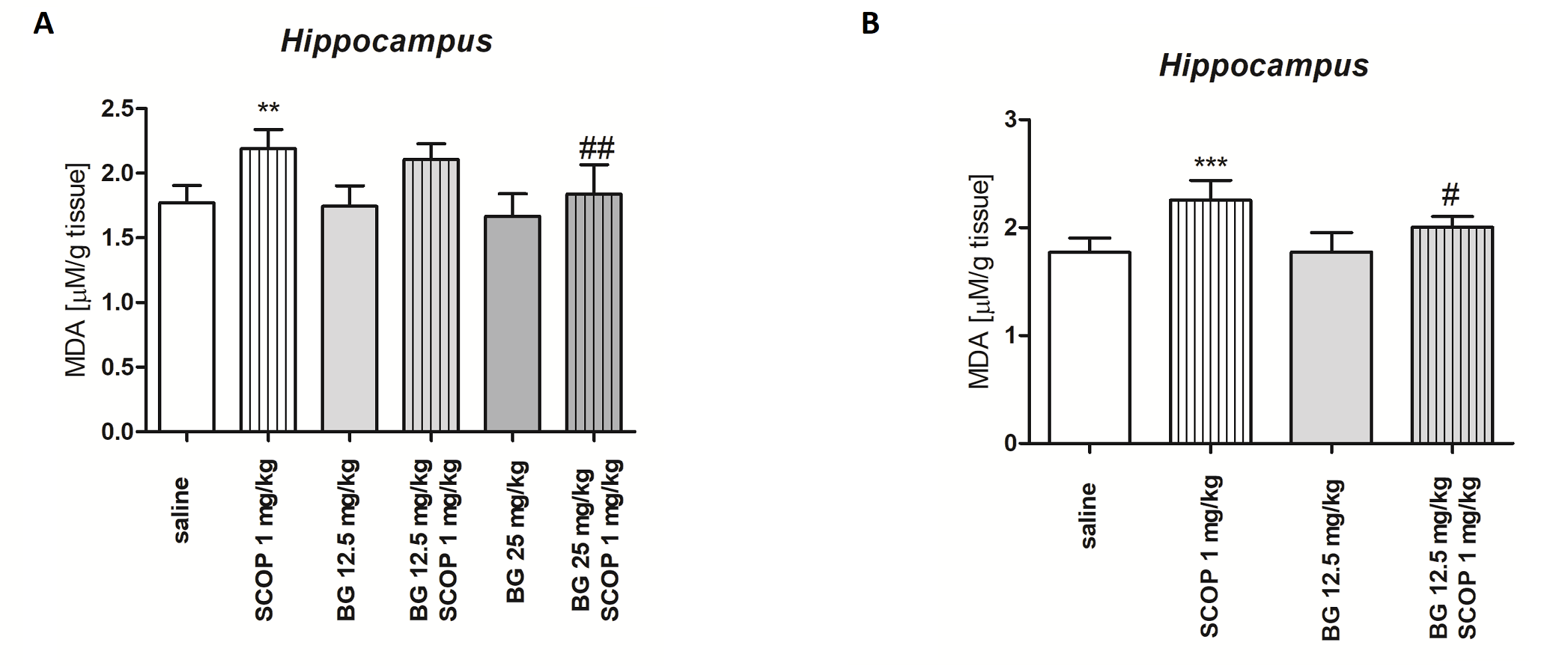

Supplement: Supplementary file 3 [file Image_3.TIF]

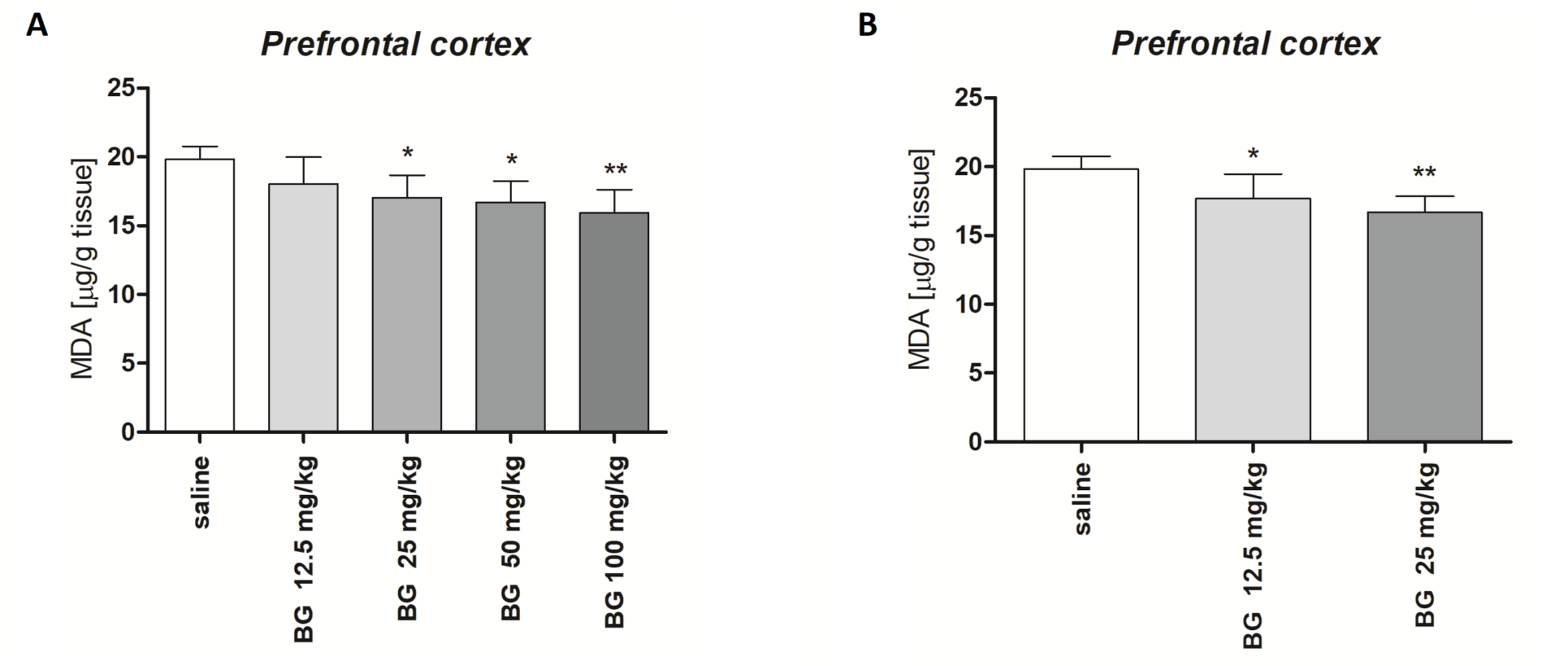

Supplement: Supplementary file 4 [file Image_4.TIF]

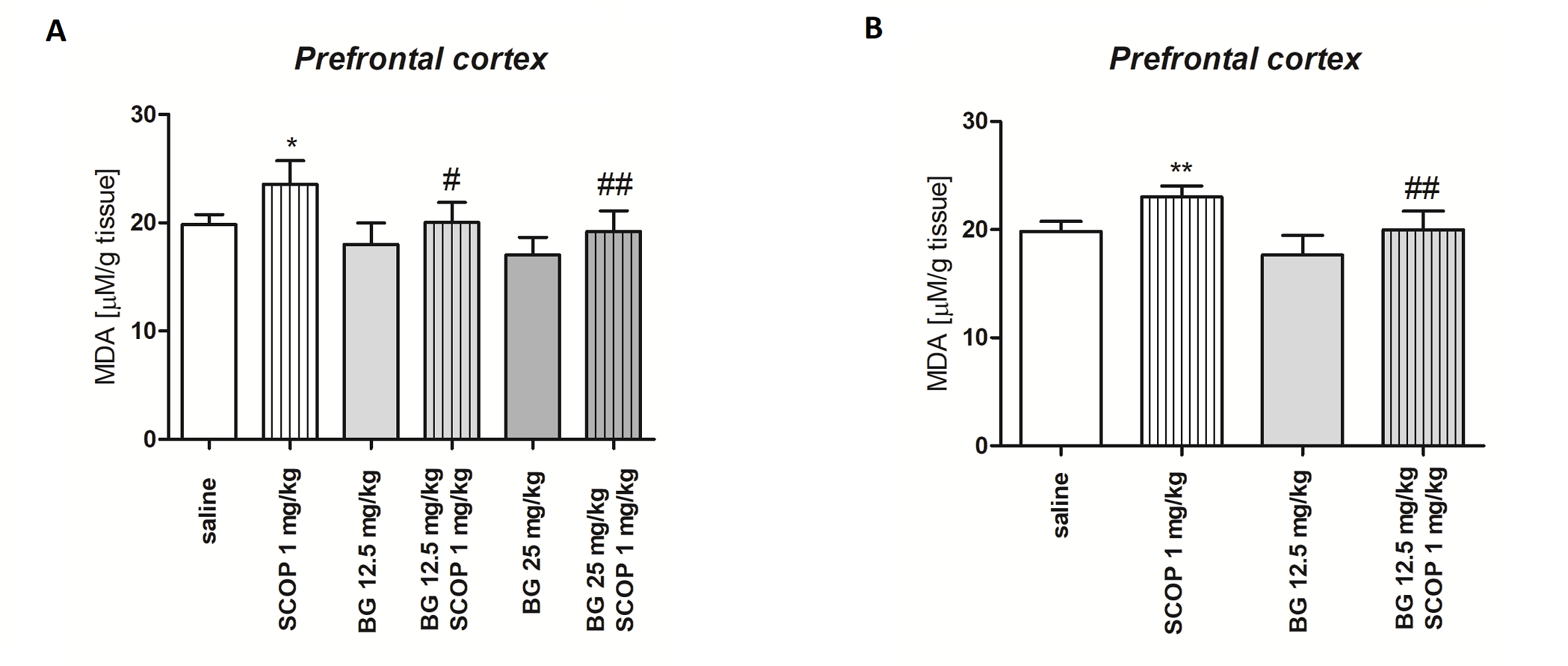

Supplement: Supplementary file 5 [file Image_5.TIF]

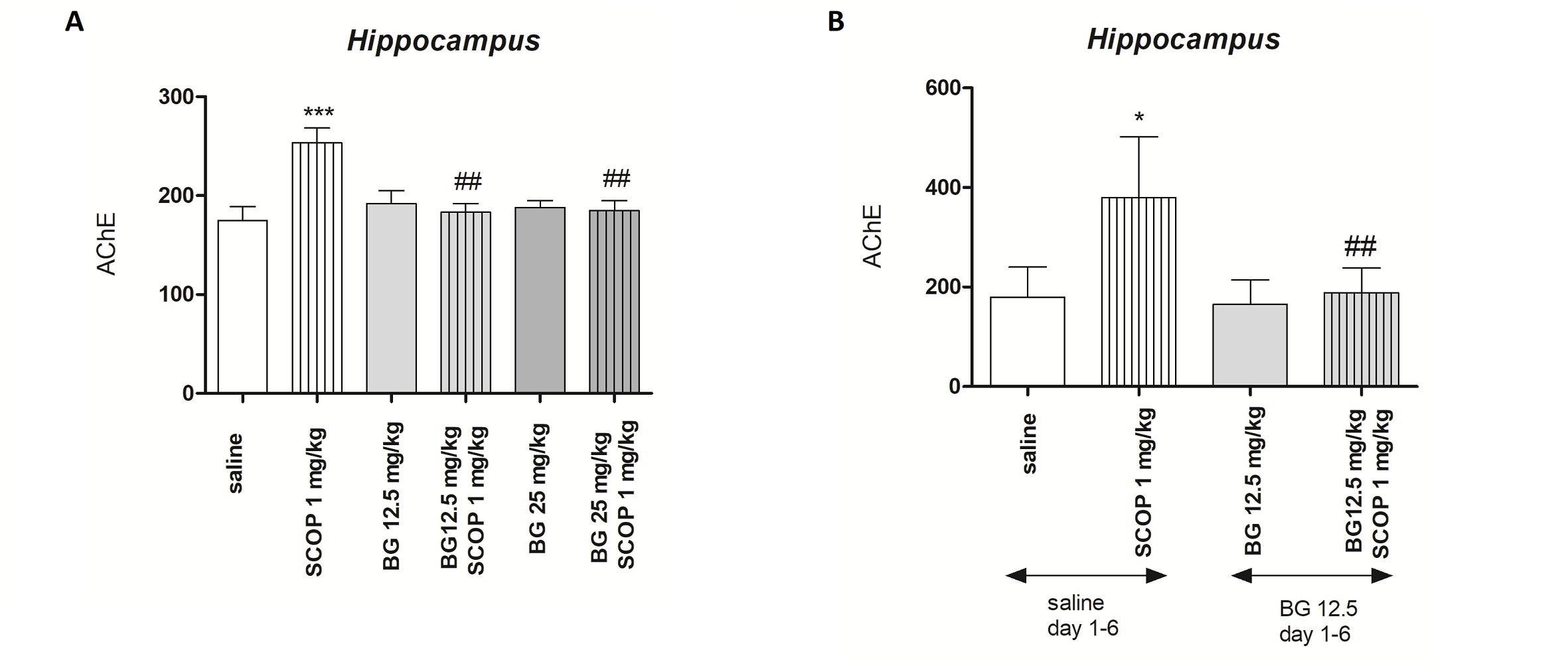

Supplement: Supplementary file 6 [file Image_6.TIF]

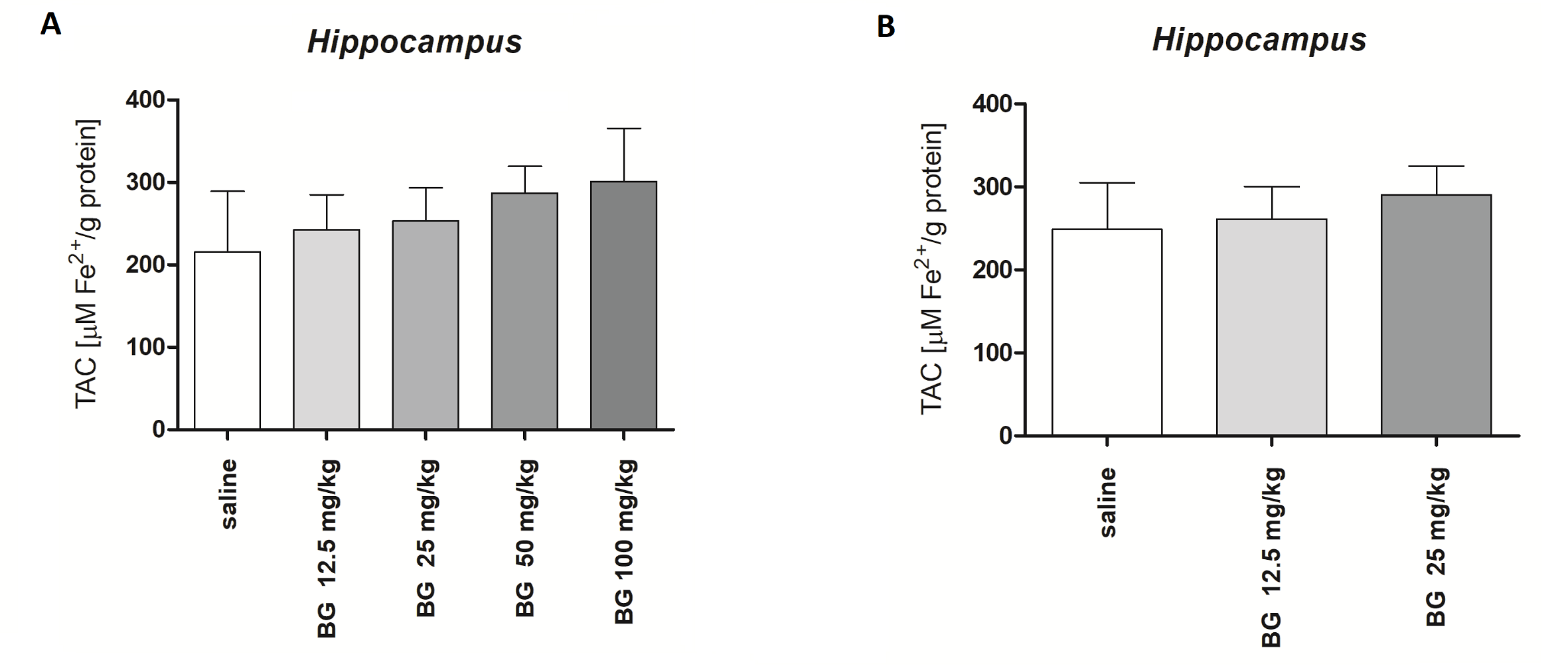

Supplement: Supplementary file 7 [file Image_7.TIF]
