# Supplementary material for: Bergapten Improves Scopolamine-Induced Memory Impairment in Mice via Cholinergic and Antioxidative Mechanisms
Source: Front Neurosci. 2020 Aug 6;14:730. doi: 10.3389/fnins.2020.00730 (PMC7438900; doi:10.3389/fnins.2020.00730)
Supplement: Supplementary file 8 [file Table_1.DOCX]

***Suplementary***

Figure 1A indicates the effects of bergapten (12.5 and 25 mg/kg) administered alone or in combination with scopolamine on AChE level measured in the hippocampus (pretreatment (F (1, 50) = 4.18, *p* = 0.0249), interactions (F (1, 50) = 9.47, *p* = 0.0003) and treatment (F (1,50) = 5.36, *p* = 0.0249); two-way ANOVA). Post hoc Bonferroni’s test confirmed the significant increasing of the AChE concentration in the hippocampus after single injection of scopolamine (*p* < 0.001). No changes were noticed after single acute injection of bergapten (12.5 and 25 mg/kg), however combination of bergapten at both doses and scopolamine decreased the level of AChE in the hippocampus (*p* < 0.01) in comparison with scopolamine-treated group (Figure [1](https://pubs.acs.org/doi/10.1021/acschemneuro.8b00011#fig5)A).

Figure 1B indicates the effects of subchronic bergapten (12.5 and 25 mg/kg) administered alone or in combination with acute injection of scopolamine on AChE level measured in the hippocampus (pretreatment (F (1, 35) = 15.20, *p* = 0.0004), interactions (F (1, 35) = 10.97, *p* = 0.0022) and treatment (F (1, 35) = 18.25, *p* = 0.0001); two-way ANOVA). Post hoc Bonferroni’s test confirmed the significant increase of the AChE concentration in the hippocampus (*p* < 0.05). No changes were noticed after single subchronic injection of bergapten (12.5 mg/kg), however combination of bergapten and scopolamine was able to completely prevent increased level of AChE induced by scopolamine the in the hippocampus (p < 0.01). (Figure [1B](https://pubs.acs.org/doi/10.1021/acschemneuro.8b00011#fig5)).

Figure 2A presents the effect of single dose of bergapten (12.5, 25, 50, 100 mg/kg) on Total Antioxidant Capacity (TAC) measured in the hippocampus (F (4, 25) = 2.932, *p =* 0.0407; one-way ANOVA). Increase in TAC concentration was observed in the hippocampus, however the values were not statistically significant (Figure 2A).

Figure 2B presents the effect of subchronic bergapten (12.5, 25 mg/kg) on TAC measured in the hippocampus (F (2, 15) = 1.404, *p =* 0.2761; one-way ANOVA). Increase in TAC concentration was observed in the hippocampus, however the values were not statistically significant (Figure 2B).

Figure 3A indicates the effect of single dose of bergapten (12.5, 25 mg/kg) administered alone or in combination with scopolamine (1 mg/kg) on TAC determined in the hippocampus (pretreatment (F (2, 54) = 10.17, *p =* 0.0002), treatment (F (1, 54) = 20.61, *p <* 0.0001) and interactions (F (2, 54) = 1.754, *p =* 0.1828); two-way ANOVA). Post hoc Bonferroni’s test confirmed statistically significant decrease in TAC level in the hippocampus after single injection of scopolamine (*p <* 0.05). Administration of bergapten caused dose-dependent increase in TAC level, being statistically significant in the hippocampus for the highest dose of bergapten (25 mg/kg, *p <* 0.05) in comparison to scopolamine-treated group (Figure 3A).

Figure 3B indicates the effect of subchronic bergapten (12.5 mg/kg) administered alone or in combination with scopolamine (1 mg/kg) on TAC determined in the hippocampus (pretreatment (F (1, 36) = 1.394*, p =* 0.2455), treatment (F (1, 36) = 12.17, *p =* 0.0013), without interactions (F (1, 36) = 0.006194, *p =* 0.9377; two-way ANOVA). No statistically significant changes were observed.

Figure 4A presents the effect of single dose of bergapten (12.5, 25, 50, 100 mg/kg) on malondialdehyde concentration (MDA) measured in the hippocampus (F (4, 25) = 0.9114, *p =* 0.4726; one-way ANOVA). No statistically significant changes were observed.

Figure 4B presents the effect of subchronic bergapten (12.5, 25 mg/kg) on MDA measured in the hippocampus (F (3, 20) = 13.61, *p <* 0.0001; one-way ANOVA). No statistically significant changes were observed.

Figure 5A indicates the effect of single dose of bergapten (12.5, 25 mg/kg) administered alone or in combination with scopolamine (1 mg/kg) on MDA concentration determined in the hippocampus (pretreatment (F (2, 54) = 10.62, *p =* 0.0001), treatment (F (1, 54) = 56.85, *p <* 0.0001), without interactions (F (2, 54) = 3.145, *p =* 0.0511); two-way ANOVA). Post hoc Tukey’s test confirmed statistically significant increase in MDA level in the after single injection of scopolamine (*p <* 0.01). Administration of bergapten caused dose-dependent decrease in MDA concentration, being statistically significant in the hippocampus for 25 mg/kg of bergapten (*p* < 0.01) in comparison to scopolamine-treated group (Figure 8).

Figure 5B indicates the effect of subchronic bergapten (12.5 mg/kg) administered alone or in combination with scopolamine (1 mg/kg) on MDA level determined in the hippocampus (pretreatment (F (1, 36) = 6.664, *p =* 0.0141), treatment F (1, 36) = 54.34*, p <* 0.0001) and interactions (F (1, 36) = 6.990, *p =* 0.0121; two-way ANOVA). Post hoc Tukey’s test confirmed statistically significant increase in concentration of MDA in the hippocampus after scopolamine injection (*p <* 0.001). Administration of bergapten caused decrease in MDA level, being statistically significant in the hippocampus (*p <* 0.05,) in comparison to scopolamine-treated group (Figure 10).

Figure 6A presents the effect of single dose of bergapten (12.5, 25, 50, 100 mg/kg) on malondialdehyde concentration (MDA) measured in the prefrontal cortex (F (4, 25) = 5.361, *p =* 0.0029; one-way ANOVA). Statistically significant decrease in MDA concentration was observed only after single injection of bergapten in doses 25 and 50 mg/kg (*p <* 0.05) and 100 mg/kg (*p <* 0.01) (post hoc Tukey test) (Figure 11).

Figure 6B presents the effect of subchronic bergapten (12.5, 25 mg/kg) on MDA measured in prefrontal cortex (F (3, 20) = 14.17, *p <* 0.0001; one-way ANOVA). Significant dose-dependent decrease in MDA level was observed after subchronic administration of MDA

Figure 7A indicates the effect of single dose of bergapten (12.5, 25 mg/kg) administered alone or in combination with scopolamine (1 mg/kg) on MDA concentration determined in the prefrontal cortex (pretreatment (F (2, 54) = 21.64, *p <* 0,0001), treatment (F (1, 54) = 32.70, *p <* 0.0001), without interactions (F (2, 54) = 1.398, *p =* 0.2558; two-way ANOVA). Post hoc Tukey’s test confirmed statistically significant increase in MDA level in the prefrontal cortex after single injection of scopolamine (*p <* 0.05). Administration of bergapten caused dose-dependent decrease in MDA concentration, being statistically significant for both doses of bergapten (12.5 and 25 mg/kg; *p <* 0.05, *p <* 0.01, respectively) in comparison to scopolamine-treated group (Figure 12).

Figure 7B indicates the effect of subchronic bergapten (12.5 mg/kg) administered alone or in combination with scopolamine (1 mg/kg) on MDA level determined in the prefrontal cortex (pretreatment (F (1, 36) = 32.99*, p <* 0.0001), treatment (F (1, 36) = 37.19, *p <* 0.0001) and interactions (F (1, 36) = 0.9092, *p =* 0.3467; two-way ANOVA). Post hoc Tukey’s test confirmed statistically significant increase in concentration of MDA in the prefrontal cortex after scopolamine injection (*p <* 0.01). Administration of bergapten caused decrease in MDA level, being statistically significant in the (*p <* 0.01, r) in comparison to scopolamine-treated group (Figure 14).

Figure legend

Figure 1. Effects of acute (A) and subchronic (B) administration of bergapten (BG) on AChE level increased by scopolamine administration in the hippocampus in mice. Bergapten was administered 30 min and scopolamine (SCOP, 1 mg/kg, *i.p*.) n = 8-12; the means ± SEM; * *p* < 0.05, *** *p* < 0.001 *vs.* saline-treated control group, ^##^ *p* < 0.01 *vs.* scopolamine-treated control group; Bonferroni’s test.

Figure 2. Total antioxidant capacity (TAC) of the hippocampus after single (A) and subchronic (B) administration of bergapten (BG). The results are expressed as micromoles per gram protein and presented as the means ± SD; n = 10;

Figure 3. Total antioxidant capacity (TAC) of the hippocampus after single (A) and subchronic (B) administration of bergapten (BG) or saline with or without scopolamine (1 mg/kg) administered one hour before the decapitation. The results are expressed as micromoles per gram protein and presented as the means ± SD; n = 10; * *p* < 0.05 vs. saline-treated control group; ^#^ *p* < 0.05 vs. scopolamine-treated group; Bonferroni’s test.

Figure 4. The concentration of malondialdehyde (MDA) in the hippocampus after single (A) and subchronic (B) administration of bergapten (BG). The results are expressed as micromoles per gram protein and presented as the means ± SD; n = 10;

Figure 5. The concentration of malondialdehyde (MDA) in the hippocampus after single (A) and subchronic (B) administration of bergapten (BG) or saline with or without scopolamine (1 mg/kg) administered one hour before the decapitation. The results are expressed as micromoles per gram protein and presented as the means ± SD; n = 10; ** *p* < 0.01, ; *** *p* < 0.001, vs. saline-treated control group; ^#^ *p* < 0.05, ^##^ *p* < 0.01 vs. scopolamine-treated group; Bonferroni’s test.

Figure 6. The concentration of malondialdehyde (MDA) in the prefrontal cortex of mice brain after single (A) or subchronic (B) administration of bergapten (BG). The results are expressed as micromoles per gram protein and presented as the means ± SD; n = 10; * *p* < 0.05, ** *p* < 0.01, vs. saline-treated control group; Tukey’s test.

Figure 7. The concentration of malondialdehyde (MDA) in the prefrontal cortex of mice brain after single (A) or subchronic (B) administration of bergapten (BG) or saline with or without scopolamine (1 mg/kg) administered one hour before the decapitation. The results are expressed as micromoles per gram protein and presented as the means ± SD; n = 10; * *p* < 0.05, ** *p* < 0.01 vs. saline-treated control group; ^#^ *p* < 0.05, ^##^ *p* < 0.01 vs. scopolamine-treated group; Bonferroni’s test.

**Measurement of Lipid Peroxidation – MDA concentration**

The level of lipid peroxidation in tissues homogenates was measured by thiobarbituric acid (TBA) test for malondialdehyde (MDA). At the beginning, a standard was prepared by hydrolysis of 16.4 μl of 1,1,3,3-tetraethoxypropane stock solution in 50 ml of 0.2 mM hydrochloric acid. Such obtained MDA standard (10 mM) was used to prepare standard curve with final concentrations of 1, 2, 3, 5, 7, and 10 μM. Then, to the homogenates were added 2.8% trichloroacetic acid (TCA) and 0.37% TBA in 50 mM NaOH. Next, the samples were placed in a boiling water bath to develop a colored MDA–TBA adduct and then centrifuged at 10,000g for 10 min. The pink chromogen was measured at 532 nm using an Epoch UV–Visible Spectrophotometer against a blank and the results were evaluated from the standard curve. As the experiment was performed in triplicate, the final results are mean values of them.
